# Identification of the serum metabolomic profile for acute ischemic preconditioning in athletes

**DOI:** 10.3389/fphys.2024.1492202

**Published:** 2024-11-06

**Authors:** Ziyue Ou, Liang Yang, Mingxin Xu, Xiquan Weng, Guoqin Xu

**Affiliations:** ^1^ College of Martial Arts, Guangzhou Sport University, Guangzhou, China; ^2^ The Fifth College of Clinical Medicine, Guangzhou University of Chinese Medicine, Guangzhou, China; ^3^ College of Exercise and Health, Guangzhou Sport University, Guangzhou, China; ^4^ Guangdong Provincial Key Laboratory of Physical Activity and Health Promotion, Guangzhou Sport University, Guangzhou, China

**Keywords:** ischemic preconditioning, metabolomic profile, taekwondo athletes, D-gluconate, pentose phosphate pathway

## Abstract

**Purpose:**

In recent years, ischemic preconditioning (IPC) has emerged as an effective strategy to increase tissue resistance against long-term ischemic damage and has been increasingly integrated into exercise regimens. However, further research is needed to explore the impact of IPC-mediated metabolic alterations from an exercise standpoint to conduct a comprehensive exploration of metabolic alterations and their exercise-related mechanisms during acute IPC.

**Methods:**

Nontarget metabolomics was performed on blood samples obtained from 8 male athletes both before and after IPC. The studies included the identification of differentially abundant metabolites, analysis of receiver operating characteristic (ROC) curves, Kyoto Encyclopedia of Genes and Genomes (KEGG) enrichment analysis for differentially abundant metabolites, and metabolite set enrichment analysis (MSEA).

**Results:**

Nineteen differentially abundant metabolites were identified, with increasing levels of five metabolites, such as O-desmethyltramadol and D-gluconate, whereas 14 metabolites, including 9-hydroxy-10e, 12z-octadecadienoic acid (9-HODE), tetradione, 2-hexenal, (2,4-dichlorophenoxy)acetic acid (2,4-D), and phosphatidylserine (PS), decreased. ROC curve analysis revealed an AUC of 0.9375 for D-gluconate. Both KEGG enrichment analysis and MSEA revealed enrichment in the pentose phosphate pathway (PPP).

**Conclusion:**

This study revealed that PPP, D-gluconate, O-desmethyltramadol, and D-2-aminobutyric acid could be upregulated within 5 min after acute IPC, whereas 2,4-D, PS, 9-HODE, 2-hexenal, and tetradinone could be downregulated. These identified metabolites show promise for improving physical functional status and could be harnessed to enhance athletic performance.

## 1 Introduction

Ischemic preconditioning is a well-established strategy that enhances tissue resilience against prolonged ischemic damage. Murry conducted repeated short-term ischemia‒reperfusion on the hearts of dogs to stimulate the body’s intrinsic protective mechanisms and reduce the degree of cardiac infarction. IPC was initially employed to safeguard the myocardium and mitigate myocardial damage resulting from prolonged ischemia (Murry et al., 1986). Additionally, it has a protective effect on the kidneys, liver, and cerebral nerves during extended periods of ischemia‒reperfusion (Choi et al., 2020; Donato et al., 2021; Yang et al., 2020; Livingston et al., 2019). Recent studies have demonstrated that IPC can enhance the performance of elite athletes by briefly and repeatedly applying sufficient pressure to the limbs to occlude blood flow, thereby inducing brief ischemia‒reperfusion (Caru et al., 2019). This process has been shown to stimulate athletic potential, leading to improvements in sports performance, including increased maximum oxygen uptake in cyclists (de Groot et al., 2010), enhanced performance in swimmers during the 100-meter race (Emilie Jean-St-Michel et al., 2011) and improved results in judo and taekwondo athletes during specialized tests (Ribeiro et al., 2019; Ou et al., 2024). These findings underscore the importance of future applications of IPC in sports training and competitions. However, the precise mechanism by which IPC enhances athletic performance remains unclear, highlighting the necessity of understanding this mechanism for the informed use of IPC in sports training to enhance performance and achieve competitive success.

Research has shown that IPC can help mitigate ischemia‒reperfusion injury through the use of adenosine, bradykinin, opioids, and other substances, along with the selective activation of mitochondrial ATP-sensitive potassium (mK_ATP_) channels (Nakano et al., 2000; Downey et al., 2007). Increased levels of mK_ATP_ and adenosine can lead to vasodilation, improve muscle blood supply (de Groot et al., 2010), and prevent vascular function decline following intense exercise (Kraemer et al., 2011). Additionally, some research has indicated that IPC can increase the maximum oxygen uptake in cyclists. Mitochondria are recognized as the primary intracellular effectors of IPC, with mK_ATP_ activation playing a critical role in cardioprotection (Heusch, 2020). IPC has the potential to stimulate muscle growth and metabolic changes, resulting in significant improvements in muscle endurance and strength in a short period, thereby enhancing athletic performance (Bailey et al., 2012; Lawson and Downey, 1993; Homer-Vanniasinkam, 2005). Recent studies have revealed that tourniquet-induced IPC can upregulate Mitofusin2 and help maintain muscle strength (Leurcharusmee et al., 2022). Furthermore, IPC can regulate microvascular dilation and exert anti-inflammatory effects through nitric oxide (NO). Improved vascular function during exercise enhances oxygen delivery efficiency, facilitates lactic acid removal, and maintains acid‒base balance (Tapuria et al., 2008). Thus, IPC can influence the body’s metabolism, with certain metabolic changes being beneficial for enhancing exercise performance. However, current research on the metabolite changes induced by IPC remains limited, necessitating further exploration of the metabolic profiles affected by IPC.

Although studies have demonstrated that ischemic preconditioning (IPC) induces relevant metabolites that enhance exercise performance, particularly aerobic activities, IPC may not universally improve performance across all forms of exercise (Caru et al., 2019). This underscores the necessity of comprehensively assessing the metabolic changes induced by IPC. Consequently, more thorough and in-depth methodologies are needed to investigate the metabolic characteristics of IPC and to elucidate its role in enhancing exercise capacity. In recent years, metabolomics research methods have gained increasing prominence in investigating mechanistic pathways by analyzing multiple metabolites in samples to uncover metabolic profiles and alterations in the body under different conditions (Jonsson et al., 2005). Nontargeted metabolomics has emerged as a valuable tool for studying the impact of IPC on the plasma metabolome. This approach can also be utilized to explore both potential and previously unidentified important metabolites. It was discovered that alpha-hydroxybutyrate could serve as a cardioprotective factor and biomarker for tissue ischemia (Laursen et al., 2017). A recent study also employed an untargeted metabolomic approach to explore the metabolic signature associated with long-term remote ischemic preconditioning, suggesting potential benefits for the diagnosis and treatment of cerebral ischemia‒reperfusion injury (Du et al., 2023). Therefore, nontargeted metabolomics technology not only facilitates a deeper understanding of the characteristics of IPC-induced metabolic profiles but also explores the potential advantages of changes in these profiles for enhancing exercise performance and identifying biomarkers related to the mechanisms of IPC.

This study hypothesized that acute IPC influences the metabolic profile of taekwondo athletes, potentially enhancing their athletic performance. The primary objective of this research was to employ nontargeted metabolomics technology to assess metabolomic changes in athletes before and after acute IPC, with an emphasis on a detailed examination of exercise-related metabolic mechanisms and pathways.

## 2 Materials and methods

### 2.1 Subjects

In light of existing research demonstrating that ischemic preconditioning can significantly enhance the performance of certain sports and considering related studies on ischemic preconditioning and metabolomics technology, we ultimately selected eight taekwondo athletes for this investigation (Baranovicova et al., 2018; Baranovicova et al., 2021; Rong et al., 2022; Wu et al., 2022; Laursen et al., 2017). The participants had to meet specific criteria to ensure their good health. The inclusion criteria were as follows: (1) adult males over 18 years of age; (2) professional athletes at the national second level; (3) a minimum of 5 years of professional training in Taekwondo; and (4) maintenance of regular and standardized training for the past 3 months. The exclusion criteria were as follows: (1) acute or chronic diseases such as anxiety, depression, cardiovascular disease, respiratory disease, or metabolic disease; (2) use of creatine supplementation; (3) recent medication use within 3 months; (4) consumption of alcohol and caffeine within the last 24 h before testing; and (5) engaging in aerobic or anaerobic exercise within the past 24 h (6) Having received ischemic preconditioning. All participants were informed about the benefits, discomfort, and potential risks associated with the study. Additionally, all participants received a thorough explanation of the study procedures, provided informed consent, and fasted for a minimum of 12 h prior to undergoing ischemic preconditioning.

This research was approved by the Ethics Review Committee of Guangzhou Sport University (ID Number: 2023LCLL-81) and conducted following the guidelines of the Declaration of Helsinki.

### 2.2 Experimental protocol of the study

**FIGURE 1:**
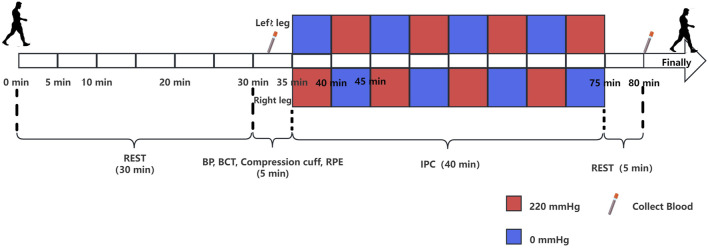
Experimental protocol for the study. The rectangle above the timeline represents the intervention for the left leg, and the rectangle below represents the intervention for the right leg. The red rectangle represents a pressure of 220 mmHg, whereas the blue rectangle represents a pressure of 0 mmHg. Each rectangle corresponds to a duration of 5 min, and the experiment proceeds from left to right.

The experimental plan consisted of several steps. The participants were instructed to abstain from alcohol, caffeine, and intense physical activity the day prior to the experiment. Upon arrival at the laboratory, the experimenter provided a detailed explanation of the procedures, obtained signed informed consent forms from the subjects, and allowed them to rest for 30 min. Subsequently, blood pressure (BP) was measured, and 5 mL blood samples were drawn from the middle cubital vein using an EDTA-K2 anticoagulated tube, which were then labeled Group A. A body composition test (BCT) was then conducted, with the participant wearing a compression cuff and lying down. Prior to the ischemic preconditioning intervention, fingertip blood was collected for analysis of lactic acid levels, along with an inquiry into the Rating of Perceived Exertion (RPE). During the second part of the experiment, the participants laid down for a total of 40 min of IPC. Following the IPC intervention, the participant remained still for an additional 5 min. At the end of this period, another blood sample (5 mL) was collected from the median cubital vein via an EDTA-K2 anticoagulant tube and labeled group B (Laursen et al., 2017). The experimental flow chart is depicted in Figure 1.

### 2.3 Basic indicator measurement methods

Blood pressure was assessed with an OMRON blood pressure meter (Model HEM-1020) via the authoritative oscillometric method (Alexandra et al., 2002). The tester held a colored rating of the Perceived Exertion scale and asked the experimental subjects, and the experimental subjects were subjectively scored according to their own feelings (Ritchie, 2012). Body composition, such as weight, fat mass and muscle mass, was measured via a body composition analyzer (Inbody 370).

### 2.4 IPC

Under normal oxygen conditions, the subject was placed in a supine position for IPC intervention. A blood pressure cuff was positioned on the upper third of both thighs (groin) of the subject. The cuff on the right leg was inflated to 220 mmHg, whereas the cuff on the left leg remained at 0 mmHg (Cheng et al., 2021; Paradis-Deschênes et al., 2020; Da Mota et al., 2019). After 5 min, the cuff on the right leg was deflated to 0 mmHg, and the cuff on the left leg was increased to 220 mmHg for another 5 min, completing one cycle. This protocol was repeated for 4 cycles, totaling 40 min.

### 2.5 Experimental methods for assessing metabolism

#### 2.5.1 Chemicals

Sigma Aldrich provided ammonium acetate (NH_4_AC), while Merck supplied acetonitrile. Fisher supplied ammonium hydroxide (NH_4_OH) and methanol.

#### 2.5.2 Sample collection and preparation

Blood was collected from the median cubital vein via EDTA-K2 anticoagulant tubes. After collection, the blood was centrifuged within 30 min at 4°C with a centrifugal force of 1,100 × g for 15 min. The serum was separated on ice boxes and frozen at −80°C.

#### 2.5.3 LC‒MS/MS analysis

Metabolites were examined via nontargeted metabolomics. Following a gradual thawing process at 4°C, a portion of the sample was extracted and combined with a chilled mixture of methanol, acetonitrile, and water at a 2:2:1 ratio. The mixture underwent vigorous mixing and ultrasonication at low temperatures for 30 min, followed by a 10-minute incubation at −20°C. The mixture was subsequently centrifuged at 14,000 × g for 20 min at 4°C, after which the liquid portion was evaporated under vacuum. For mass spectrometry analysis, the dehydrated sample was reconstituted with 100 μL of an aqueous solution of acetonitrile (acetonitrile:water = 1:1), followed by mixing and centrifugation at 14,000 × g for 15 min at 4°C. The liquid portion was then used for sample analysis. Quality control (QC) samples were prepared by extracting 10 μL from each sample, with a QC sample analysis performed after every five samples were analyzed. The primary and secondary spectra were collected via an AB Triple TOF 6600 mass spectrometer. After HILIC separation, the ESI source conditions included gas settings such as Ion Source Gas1 (Gas1): 60, Ion Source Gas2 (Gas2): 60, and Curtain gas (CUR): 30. The source temperature was set at 600°C, and the IonSapary Voltage Floating (ISVF) was ±5500 V for both positive and negative modes. For mass spectrometry analysis, the TOF MS scan ranged from 60 to 1,000 Da, whereas the product ion scan ranged from 25 to 1,000 Da. The accumulation time for the TOF MS scan was 0.20 s/spectra, and that for the product ion scan was 0.05 s/spectra. The secondary mass spectrum was obtained via information-dependent acquisition (IDA) in high-sensitivity mode, with settings including a declustering potential (DP) of ±60 V for both positive and negative modes, a collision energy of 35 ± 15 eV, and the exclusion of isotopes within 4 Da. Moreover, the number of candidate ions to monitor per cycle was set at 10.

### 2.6 Data analysis

The raw mass spectrometry data were first converted to MzXML files via ProteoWizard MSConvert and then imported into the freely available XCMS software. Peak picking was performed with centWave using a mass‒charge ratio tolerance of 10 ppm, a peak width ranging from 10 to 60, and prefilter values between 10 and 100. For peak grouping, the bandwidth was set to 5, the mzwid value was 0.025, and the minfrac value was 0.5. The Collection of Algorithms of MEtabolite pRofile Annotation was used for annotating isotopes and adducts. Only variables with more than 50% nonzero measurement values in at least one group were retained in the extracted ion features. Metabolite compound identification involved comparing accurate mass‒charge ratio values (within 10 ppm) and MS/MS spectra with an in-house database of authentic standards. Missing data points were imputed via the K-nearest neighbor (KNN) method, and extreme values were removed. Finally, the total peak area data were normalized to ensure consistency across samples and metabolites.

Positive ion mode (POS) and negative ion mode (NEG) were utilized for metabolite detection. The data were subjected to systematic clustering analysis (Yuan et al., 2012), and the resulting dendrogram was generated via average linkage. Hierarchical clustering was conducted with the R package pheatmap (Kolde, 2015).

The screening criteria for identifying differentially abundant metabolites included a variable importance for the projection (VIP) of orthogonal partial least squares discriminant analysis (OPLS-DA) ≥1 and a significance level of *P* < 0.05 in the single-factor t-test (Han et al., 2022; Zhu et al., 2022; Deng et al., 2021). Additionally, cross-validation of the OPL-DA model was performed. Fold changes (FCs) between the two groups were calculated, and a volcano plot was generated. A chart displaying the top 15 metabolites with the highest VIP scores from OPLS-DA was created (Yoon et al., 2020). To quantify metabolite composition and abundance variability among samples, correlation analysis of sample data was performed (Rao et al., 2016). Correlations were calculated via the R corrploy package (Wei T, 2017), and a heatmap was generated via the pheatmap package. A correlation coefficient approaching 1 indicates greater similarity in metabolic composition and abundance among samples.

Abundance normalization of differentially abundant metabolites was performed via the Z score, and hierarchical clustering with the R package pheatmap was employed (Kolde, 2015). The differentially abundant metabolites were then hierarchically clustered, samples were grouped, and a cluster heatmap was generated to visualize the cumulative difference between the two groups.

Receiver operating characteristic curve analysis was conducted via the R pROC package to assess the predictive ability of each discriminant metabolite. The AUC was determined by numerically integrating the ROC curves. The area under the ROC curve (AUC-ROC) was determined via the bootstrapping method to estimate the median and 95% confidence interval (95% CI).

The calculation formula for differentially abundant metabolite KEGG enrichment analysis is as follows:

$$P=1-\sum_{i=0}^{m-1}\frac{\left(\begin{array}{c} M\\ i \end{array}\right)\left(\begin{array}{c} N-M\\ n-i \end{array}\right)}{\left(\begin{array}{c} N\\ n \end{array}\right)}$$

N represents the total count of metabolites labeled under the KEGG database, whereas n denotes the count of metabolites showing differential expression within N. Similarly, M represents the total count of metabolites labeled under a specific pathway, and m represents the count of metabolites showing differential expression within M. The p-value calculated underwent false discovery rate (FDR) correction, and a threshold of FDR ≤ 0.05 was applied. Pathways satisfying these criteria were considered significantly enriched in differentially abundant metabolites.

Metabolic set enrichment analysis (Xia and Wishart, 2010) was applied to assess pathway overrepresentation with the MetaboAnalyst module. To conduct the analysis, the Small Molecule Pathway Database library was utilized. The overrepresentation analysis was performed via Fisher’s exact test with the R package MSEAp (https://rdrr.io/github/afukushima/MSEAp/).

## 3 Results

### 3.1 Subjects

Basic characteristics of the 8 athletes before the intervention (Table 1).

**TABLE 1:** Basic information of the study subjects.

	Age (Y)	Height (cm)	Weight (kg)	Years of training (Y)	BMI (kg/m^2^)	Body fat percentage (%)	RPE
$\bar{X}\pm S$	20.25 ± 1.83	178.38 ± 5.32	71.64 ± 9.92	6.88 ± 1.36	22.45 ± 2.40	13.86 ± 6.17	6.25 ± 0.70

### 3.2 Data quality control and metabolite statistical results

**FIGURE 2:**
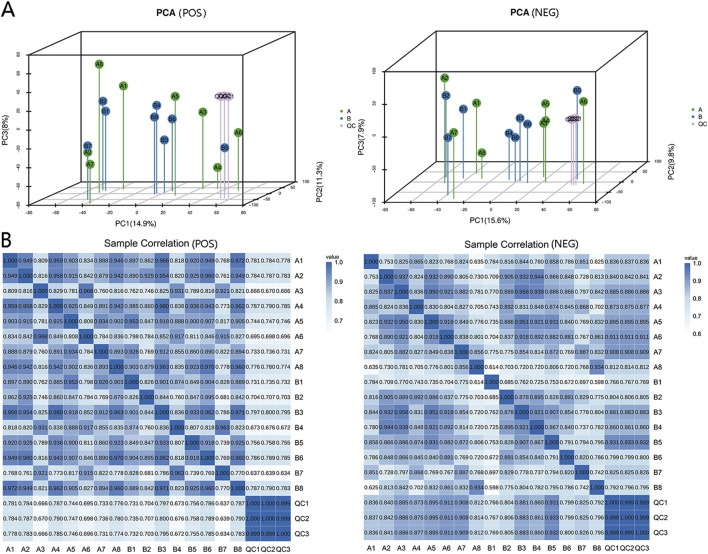
Data quality control and sample correlation. **(A)** Three-dimensional PCA plots demonstrating the metabolic variations within and between sample groups. Enhanced method stability and data quality are indicated by minimal differences among QC samples. The dense distribution of QC samples on the three-dimensional PCA chart ensures data reliability. **(B)** Sample correlation analysis was used to quantify and assess alterations in metabolite composition and abundance across samples via correlation data. A correlation value approaching 1 signifies a strong resemblance in metabolic composition and abundance among samples.

A total of 11,137 metabolites were identified in positive ion mode, comprising 1,468 known metabolites and 9,669 unknown metabolites. In negative ion mode, 9,001 metabolites were detected, with 789 known and 8,212 unknown metabolites. We labeled the blood samples before and after IPC as Group A and Group B, respectively. Principal component analysis (PCA) was performed on metabolites from Group A, Group B, and QC samples, resulting in a three-dimensional PCA diagram (Figure 2A). The analysis of the samples in the QC group demonstrated high stability, good data quality, and reliable data analysis. Additionally, a heatmap illustrating positive and negative ion correlations among samples was generated via Pearson correlation coefficients (Figure 2B).

### 3.3 Differential expression of serum metabolites

The serum metabolomic characteristics of the experimental subjects were analyzed via LC‒MS/MS before and after ischemic preconditioning. Group A represents the serum before IPC, whereas group B represents the serum after IPC. Differentially abundant metabolites were screened via both positive and negative ion modes simultaneously, and 3D PCA plots were generated for both modes (Figure 2A).

**FIGURE 3:**
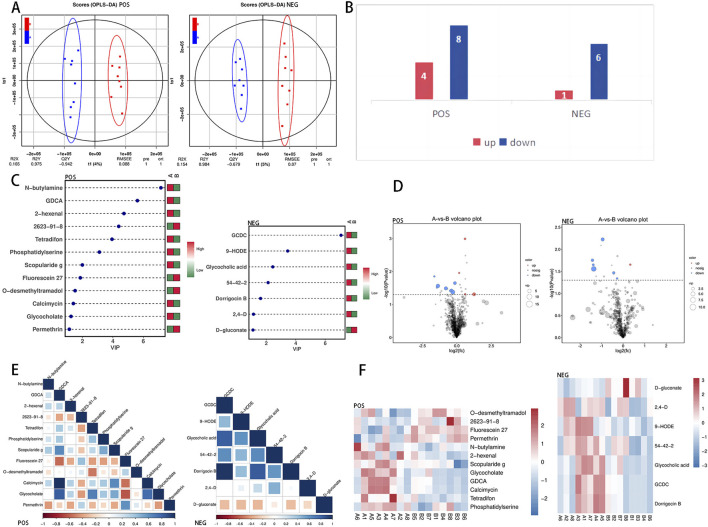
Differentially abundant metabolite identification. **(A)** Combining orthogonal signal correction (OSC) and PLS-DA simplifies the model, enhances explanatory power, and maintains predictive ability. This method optimizes the ability to distinguish between Group **(A)** and Group **(B)**. The VIP value in OPLS-DA is used to identify important metabolites, with a VIP value greater than 1 indicating significance. **(B)** Statistical chart of differential metabolism upregulation and downregulation in positive and negative ion modes. **(C)** The x-axis shows the VIP value, the y-axis lists the differentially abundant metabolites, and the color legend indicates the abundance in different groups; red indicates upregulation, and green indicates downregulation. A higher VIP value indicates a stronger contribution to group distinction, with metabolites having a VIP value above 1 showing significant differences. **(D)** The x-axis depicts the log2-fold difference in metabolite abundance for each control group, whereas the y-axis illustrates the −log10 of the P-value after the T-test. The dashed line perpendicular to the y-axis indicates the P-value threshold for screening differentially abundant metabolites. Red dots indicate upregulated differentially abundant metabolites (FC > 1) with VIP ≥ 1 and *P* < 0.05, whereas blue dots represent downregulated differentially abundant metabolites (FC < −1) with VIP ≥ 1 and *P* < 0.05. The size of the dots corresponds to the VIP value of the metabolite. **(E)** Differentially abundant metabolite correlation heatmap. Positive correlations are depicted by dark blue shading, which approaches 1, whereas negative correlations are shown by dark red shading, which approaches −1. The color gradient below the heatmap illustrates the Pearson correlation coefficient between the two differentially abundant metabolites. **(F)** In the heatmap, each row represents a metabolite, and each column represents a sample. The color intensity reflects the abundance of the metabolite, with red indicating higher abundance and blue indicating lower abundance. The differentially abundant metabolites presented diverse accumulation patterns between group **(A)** and group **(B)**.

The analysis revealed that there were no significant differences in the first, second, or third principal components among the samples from Group A and Group B; however, Group B presented a greater level of clustering than did Group A (Figure 2A). Differentially abundant metabolites between the two groups were identified via an OPLS-DA model, and positive and negative ion OPLS-DA score plots were generated (Figure 3A). The cross-validation results of the OPLS-DA model indicate that in positive ion mode, R2Y = 0.975, whereas in negative ion mode, R2Y = 0.984, demonstrating the model’s high interpretability. A total of 19 differentially abundant metabolites were identified on the basis of the criteria of VIP ≥1 in the OPLS-DA and *P* < 0.05 in the t-test of univariate statistical analysis. Among these metabolites, 5 presented increased levels in group B, including O-desmethyltramadol, D-2-aminobutyric acid, and D-gluconate, whereas 14 metabolites presented decreased levels, such as N-butylamine, (2,4-dichlorophenoxy) acetic acid, 9-hydroxy-10e, 12z-octadecadienoic acid, tetradifon, PS, glycocholic acid, calcimycin, glycodeoxycholic acid (GDCA), and 2-hexenal. A statistical chart illustrating the differentially abundant metabolites associated with positive and negative ions was also generated (Figure 3B).

To clearly display the differentially abundant metabolites, we calculated the VIP values of the metabolites and generated an OPLS-DA VIP statistical chart (Figure 3C). Among these metabolites, glycochenodeoxycholate (GCDC) presented the highest VIP value, indicating its significant role in sample differentiation. We subsequently calculated the fold change in the abundance of the metabolites and created a volcano plot on the basis of the VIP and P values (Figure 3D). Pearson correlation coefficient analysis was conducted to examine the relationships among the metabolites, leading to the generation of a heatmap illustrating their correlations (Figure 3E). Furthermore, the differentially abundant metabolite clustering heatmap results (Figure 3F) highlighted distinct accumulation patterns among the comparison groups, demonstrating a clear aggregation trend.

**FIGURE 4:**
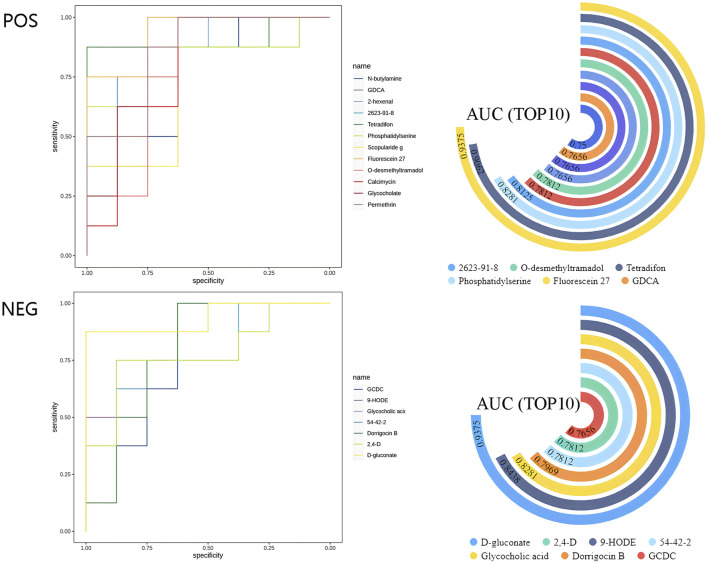
ROC analysis of differentially abundant metabolites. The ROC curve analysis on the left was utilized to assess the accuracy of the metabolites as biomarkers. The x-axis represents the 1-specificity value, whereas the y-axis represents the sensitivity value. The AUC value, which indicates the area under the curve, is employed to determine the accuracy of a species as a biomarker. AUC = 0.5 ∼ 0.7 signifies lower accuracy, AUC = 0.7 ∼ 0.9 indicates moderate accuracy, and higher accuracy is achieved with AUC values above 0.9. The circle plot on the right displays the top 10 differentially abundant metabolites with corresponding AUC values. Each circle represents a specific metabolite, and the proximity of the circle to 270° indicates a higher AUC value closer to 1.

Receiver operating characteristic curve analysis was conducted on the differentially abundant metabolites. The statistical chart (Figure 4) displaying the ROC curve and AUC values revealed that the AUC for the differentially abundant metabolite D-gluconate was 0.9375, which was the highest among all the differentially abundant metabolites.

### 3.4 Pathway analysis

#### 3.4.1 KEGG annotations and enrichment analysis of differentially expressed metabolites

The Kyoto Encyclopedia of Genes and Genomes (Ogata et al., 1999) serves as the primary public database for pathways, identifying key biochemical metabolism and signal transduction pathways involving metabolites. This resource is valuable for conducting metabolic analysis and researching metabolic networks in organisms, allowing researchers to examine metabolites and their expression information within a comprehensive network.

**FIGURE 5:**
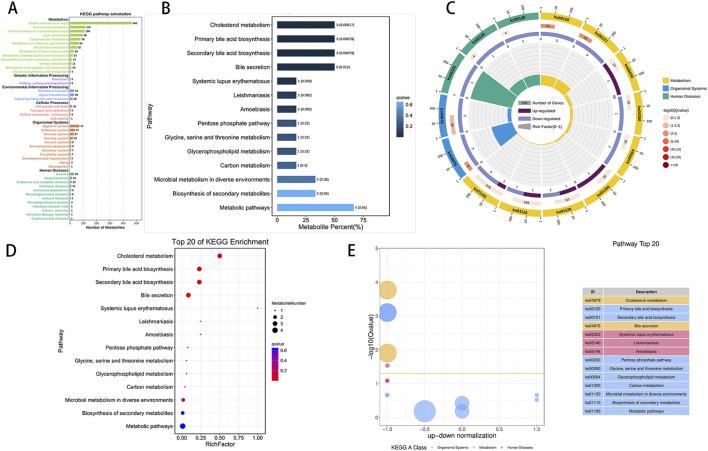
KEGG enrichment analysis of differentially abundant metabolites. **(A)**: Visualization of the distribution of metabolites across various KEGG pathway classifications, highlighting metabolism as the most prevalent category. The x-axis denotes the number of metabolites, whereas the y-axis indicates pathway classification. **(B)**: The ordinate represents the pathway, and the abscissa shows the percentage of pathways as a proportion of all differentially abundant metabolites. Darker colors indicate smaller Q values, with each column displaying the number of pathways and their corresponding Q value. A Q value less than 0.05 after multiple testing correction indicates significant pathway enrichment. The Q value represents the p-value after FDR correction. **(C)**: The KEGG enrichment circle plot shows differentially abundant metabolites in different pathways. The first circle depicts the enriched pathway, with an external coordinate ruler indicating the number of differentially abundant metabolites. Various colors represent distinct KEGG A classes. The second circle represents the number of pathways and Q values in the background, with longer bars indicating more differentially abundant metabolites and redder colors indicating smaller Q values. The third circle presents a bar chart displaying the proportion of up- and downregulated metabolites, with dark purple denoting upregulated metabolites and light purple denoting downregulated metabolites. The fourth circle illustrates the RichFactor value for each pathway, with grid lines representing increments of 0.1. **(D)**: This image displays a KEGG enrichment bubble chart illustrating enriched pathways. The y-axis represents pathways, whereas the x-axis represents the enrichment factor (the ratio of differentially abundant metabolites in the pathway to all quantities in the pathway). The size of the bubbles indicates the significance, with redder colors indicating smaller Q values. **(E)**: The chart depicts differences in KEGG enrichment, with the y-axis showing −log10 (Q value) and the x-axis representing the z score value (the difference between upregulated and downregulated metabolites as a proportion of total differentially abundant metabolites). The yellow line signifies a Q value threshold of 0.05. The right side of the image displays the top 20 pathways on the basis of the Q values, with different colors representing different classes.

The KEGG statistical plot results of all the metabolites (Figure 5A) indicated that a total of 1,067 metabolites were functionally associated with metabolism. Subsequently, KEGG enrichment analysis identified six candidate differentially abundant metabolites with pathway annotations: glycocholate, glycocholic acid, GCDC, PS, D-gluconate, and 2,4-D. Following ischemic preconditioning, a total of 14 enriched pathways were identified. These pathways include cholesterol metabolism (hits: glycocholate, glycocholic acid, and GCDC), primary bile acid biosynthesis (hits: glycocholate, glycocholic acid, and GCDC), secondary bile acid biosynthesis (hits: glycocholate, glycocholic acid, and GCDC), bile secretion (hits: glycocholate, glycocholic acid, and GCDC), systemic lupus erythematosus (hits: PS), leishmaniasis (hits: PS), the pentose phosphate pathway (hits: D-gluconate and PS), glycine, serine and threonine metabolism (hits: PS), glycerophospholipid metabolism (hits: PS), carbon metabolism (hits: D-gluconate), microbial metabolism in diverse environments (hits: D-gluconate and 2,4-D), biosynthesis of secondary metabolites (hits: D-gluconate and PS), and metabolic pathways (hits: D-gluconate, glycocholate, glycocholic acid, and PS), among which the first five pathways are significantly enriched. Consequently, a KEGG enrichment bar chart of differentially abundant metabolites was generated (Figure 5B). Pathway enrichment analysis was further conducted, resulting in the creation of a differentially abundant metabolite enrichment circle diagram (Figure 5C). The KEGG enriched pathway bubble plot (Figure 5D) highlights cholesterol metabolism as the pathway with the most significant differentially abundant metabolite enrichment among all the obtained pathways. Additionally, the differentially abundant metabolite D-gluconate, enriched in the pentose phosphate pathway, was upregulated. Furthermore, KEGG enrichment difference analysis (Figure 5E) was conducted to illustrate the enrichment differences in each pathway.

Although six differentially abundant metabolites were annotated by enriched pathways, there are still unannotated metabolites, such as 2-hexenal, D-2-aminobutyric, O-desmethyltramadol, calcimycin, 2,4-D, and 9-HODE. These substances may indicate important changes that have occurred or are about to occur after IPC. Furthermore, the metabolites annotated in this study may play other significant roles in chronically trained taekwondo athletes. Therefore, we further explored the metabolomic characteristics of these differentially abundant metabolites related to exercise in chronically trained taekwondo athletes.

#### 3.4.2 Metabolic set enrichment analysis

**FIGURE 6:**
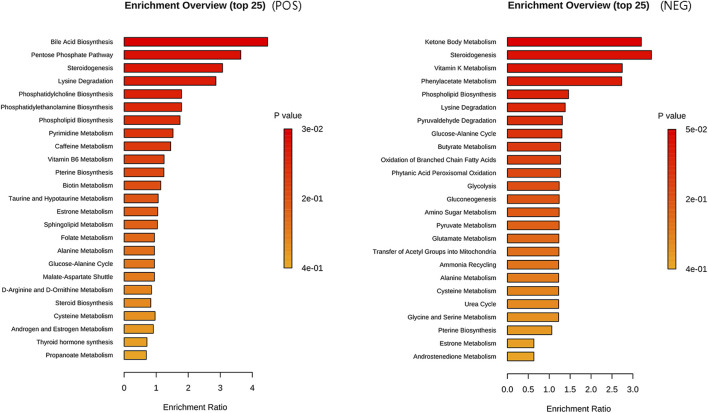
Metabolite set enrichment analysis plot. The enriched pathways in the metabolic set are listed on the left side. The length of each column represents the degree of enrichment, whereas the color indicates the p-value. Please sort the pathways according to the p-value, from smallest to largest.

Our results revealed statistical significance (*P* < 0.05) for bile acid biosynthesis and the pentose phosphate pathway in the metabolite set enrichment analysis, and the top 25 enriched pathways are visually represented in Figure 6.

## 4 Discussion

Our study revealed that PPPs can be enriched within 5 minutes following IPC, providing a reference for the potential use of IPC as an intervention to increase sports performance and its onset time. Additionally, our research clarified the effectiveness of IPC-induced PPP enrichment in human experiments. Furthermore, D-gluconate, identified as a potential biomarker of IPC, can be utilized to ascertain whether the PPP-related protective mechanism of IPC is activated by measuring the concentration of D-gluconate. IPC may reduce the body’s reliance on synthesizing ribose-5-phosphate 1-pyrophosphate (PRPP) via the oxidative pentose phosphate pathway (OPPP) through the upregulation of d-gluconate, which aids in ATP replenishment and the scavenging of accumulated free radicals. Metabolite analysis, which revealed changes in O-desmethyltramadol, D-2-aminobutyric, 2,4-D, PS, 9-HODE, 2-hexenal, and tetradenal, provides evidence supporting the potential of IPC to increase fatigue resistance and neutralize free radicals.

In our investigation, we observed a significant enrichment of the PPP in athletes undergoing IPC, which aligns with previous findings. However, earlier studies reported significant PPP activation 24 h after IPC and ischemia‒reperfusion interventions in rats (Geng et al., 2019). In contrast, our results demonstrated that the PPP was already enriched in athletes just 5 min after IPC. This discrepancy may stem from limitations in the experimental sampling time, which could have restricted the detection of PPP activation in earlier studies. Furthermore, another study indicated that PPP was enriched during specific tests in Taekwondo athletes following IPC. This further suggests that PPP may be a potential mechanism by which IPC enhances athletic performance (Ou et al., 2024). Consequently, the enrichment of PPP after IPC implies that IPC may be utilized as an intervention method to improve exercise performance while also clarifying the effectiveness of PPP enrichment in human experiments post-IPC. Simultaneously, our study offers a reference for the optimal onset time of IPC. Additionally, ROC analysis revealed that the AUC value of the differentially abundant metabolite D-gluconate was 0.9375, identifying it as a promising biomarker for IPC (Wei et al., 2011). The detection of D-gluconate may provide an effective method for rapidly assessing the success of IPC by reflecting the activation of the PPP. This aspect has rarely been mentioned in previous studies, suggesting that it may hold great significance in the practical application of IPC in sports training and warrants further research.

Notably, the IPC-induced upregulation of D-gluconate, along with the increased activation of the PPP, may contribute to increased antioxidant capacity in the body. This study underscores the importance of both the PPP and D-gluconate in the context of IPC, as demonstrated through metabolite analysis, KEGG enrichment analysis, and MSEA. The upregulation of the PPP and D-gluconate contributes to dihydronicotinamide-adenine dinucleotide phosphate (NADPH) and reactive nitrogen species (RNS) generation (Teslaa et al., 2023), such as NO, enhancing the body’s resistance to oxidative stress and preventing free radical generation (Kloska et al., 2022). This has a positive impact on endurance sports performance (Reid, 2016a; Reid, 2016b; Henríquez-Olguín et al., 2019). Furthermore, NO can enhance exercise performance by improving oxygen delivery, energy efficiency, contractility, and endurance levels (Reid, 2016b). NADPH oxidase plays a crucial role in IPC, offering protection against exercise-induced tachycardia (Sánchez et al., 2008; Bell et al., 2005). A study examining the effects of IPC intervention on the upper limbs in relation to swimming performance suggested that IPC could trigger the release of a protective factor and alter skeletal muscle tolerance to intense exercise (Emilie Jean-St-Michel et al., 2011), ultimately increasing maximum exercise capacity and endurance. This aligns with our own research findings.

Interestingly, an increase in plasma D-gluconate levels was observed, which may play a role in maintaining energy balance and preparing for a sustained energy supply. Research has indicated that the limited capacity of the oxidative pentose phosphate pathway (OPPP) in bodily tissues constrains the production of ribose 5-phosphate (R5P), leading to an inadequate supply of ribose phosphate pyrophosphate pools. Consequently, this hinders the synthesis of adenine nucleotides, hampering ATP generation and restoration in tissues and causing the accumulation of free radicals, which is particularly noticeable in muscle tissue (Zimmer et al., 1990; Zimmer et al., 1973; Zimmer, 1992; Perl et al., 2011). Notably, our study revealed significant upregulation of the differentially abundant metabolite D-gluconate in athletes following ischemic preconditioning. D-gluconate can be converted to 6-phospho-D-gluconate by gluconokinase, and through subsequent catalysis by 6-phosphogluconate dehydrogenase and ribose 5-phosphate isomerase A, it bypasses the OPPP pathway to generate R5P, enhancing PRPP synthesis and ultimately replenishing bodily tissue ATP. This finding aligns with prior research (Murry et al., 1986). Moreover, studies suggest that supplementing ribose *in vitro* can alleviate or prevent a decrease in ATP by bypassing the OPPP pathway (Zimmer, 1992). Hence, the increase in D-gluconate levels can effectively restore ATP in bodily tissues without relying on the OPPP pathway, thereby sustaining energy equilibrium. This phenomenon holds particular significance for skeletal muscle, where the OPPP pathway is most constrained (Zimmer et al., 1990). Although our study did not include exercise performance testing, the observed upregulation of D-gluconate and enrichment of the pentose phosphate pathway suggest that the mechanism by which ischemic preconditioning enhances exercise performance may be related to these factors. Consequently, further targeted research is warranted.

The upregulation of the PPP and D-gluconate is crucial for the body’s redox system and energy balance. However, the functional properties of other plasma metabolites that are advantageous for exercise should not be overlooked. This study aligns with previous research demonstrating that IPC triggers the production of endogenous opioids in experimental subjects. O-desmethyltramadol, an opioid, is notably increased after ischemic preconditioning, leading to cardioprotection through the stimulation of d1-opioid (Schultz et al., 1998a) receptors and the activation of protein kinase C (PKC) (Fryer et al., 1999; Schultz et al., 1998b; Ytrehus et al., 1994). Research indicates that PKC and NO (produced from NADPH) can increase mK_ATP_ levels (Sato et al., 2000). Elevated mK_ATP_ levels promote vasodilation, preventing vascular function decline after high-intensity exercise and increasing blood flow to muscles (Kraemer et al., 2011; Enko et al., 2011). Additionally, NO has vasodilatory effects, improving blood oxygen delivery capacity (Moncada et al., 1991). Enhanced vascular function during exercise facilitates oxygen transport, lactic acid removal, maintenance of acid‒base balance, and overall body function (Cooper and Brown, 2008). O-desmethyltramadol also provides analgesic effects by inhibiting norepinephrine reuptake and stimulating serotonin release (Poulsen et al., 1996; Patel et al., 2009). The heightened levels of O-desmethyltramadol seem to alleviate acute pain associated with training, enabling athletes to fully execute their skills and tactics. Moreover, the metabolite D-2-aminobutyric acid significantly increased, potentially increasing intracellular glutathione levels through AMPK activation and offering protection against oxidative stress. Research has indicated that D-2-aminobutyric acid supplementation can increase myocardial and circulating glutathione levels, preventing doxorubicin-induced cardiomyopathy in mice (Irino et al., 2016). Consequently, ischemic preconditioning leads to the upregulation of O-desmethyltramadol and D-2-aminobutyric acid in plasma, which aids in vasodilation, enhances antioxidant capacity, improves oxygen transport, increases lactate clearance, and alleviates the negative effects of high-intensity exercise, particularly endurance training. This process also helps maintain vascular function, offering protective effects on the cardiovascular system and analgesic benefits. The metabolite changes induced by ischemic preconditioning seem to enhance the performance of athletes.

In addition, the plasma levels of the differentially abundant metabolites 2,4-D, PS, 9-HODE, 2-hexenal, and tetradenal were notably reduced following IPC. 2,4-D is a superoxide-producing oxidant that increases the expression of PPP metabolites and G6PD in dopaminergic cell lines, inducing oxidative stress and neurotoxicity (Zhangxue et al., 2012; Higuchi et al., 2001). In this study, the downregulation of 2,4-D content resulting from IPC helped prevent the development of movement disorders (Tu et al., 2019). Exogenous PS supplementation has been shown to mitigate the emergency response of serum cortisol and creatine kinase to acute exercise (Monteleone et al., 1990; Monteleone et al., 1992; Fernholz, 2000), maintaining skeletal muscle movement under high-intensity exercise or hypoxic conditions, prolonging the time to fatigue, and ultimately improving exercise performance (Schumacher et al., 2021; Kingsley et al., 2005; Kingsley Mi et al., 2006). However, in our experiment, PS levels decreased, indicating its utilization in coping with emergency responses during IPC and activating relevant mechanisms through preadaptation, which helps to protect the exercise capacity of skeletal muscles. 9-HODE, a bioactive oxidized linoleic acid metabolite, is associated with oxidative stress and the inflammatory response in the body (Spiteller and Spiteller, 1997; Nieman et al., 2014). Research indicates that 9-HODE serves as an oxidative stress biomarker following acute exercise (Nieman et al., 2014). The reduction in 9-HODE after IPC suggests that IPC enhances the body’s capacity to combat oxidative stress, thereby potentially improving exercise performance. Additionally, a reduction in 2-hexenal in the blood can lower the production of ROS, whereas decreased tetradinone levels can help prevent mutagenesis (Moriya et al., 1983) and damage the antioxidant system (Badraoui et al., 2007). The notable decrease in these plasma metabolites mediated by IPC suggests an increase in the body’s antioxidant capacity and a reduction in the body’s inflammatory response. All these factors are conducive to enhanced exercise performance.

The results revealed significant enrichment in cholesterol metabolism, primary bile acid biosynthesis, secondary bile acid biosynthesis, and bile secretion, with a decrease in the number of annotated differentially abundant metabolites. This contrasts with findings from a study on the metabolomic characteristics of long-term IPC training (Du et al., 2023), suggesting potential adaptive changes in the body following prolonged training. While reduced GCDC and Calcimycin levels were shown to aid in preventing hepatocyte ATP depletion and subsequent Ca^2+^ increase, thereby reducing toxicity to hepatocytes (Spivey et al., 1993; Pressman, 1976), changes in these substances have not been reported in studies on long-term IPC training. Notably, the PPP enrichment observed in this study has not been documented in long-term IPC training research. This discrepancy may stem from the differing mechanisms underlying acute IPC and long-term IPC, warranting further investigation.

## 5 Conclusion

Nontarget metabolomics technology is effective for identifying the metabolic profiles associated with acute IPC. Within 5 min following acute IPC, the levels of PPP, D-gluconate, O-desmethyltramadol, and D-2-aminobutyric acid were upregulated, whereas 2,4-D, PS, 9-HODE, 2-hexenal, and tetradenal were downregulated. Additionally, D-gluconate has the potential to serve as a biomarker for IPC, facilitating the rapid detection of IPC activation status. These changes in metabolic profiles and metabolic pathways may be the potential mechanism by which IPC improves exercise performance. However, this study primarily examined metabolic changes in male athletes, highlighting the need for further research to explore sex differences. Additionally, the investigation was limited to metabolic changes occurring within 5 min following IPC, without assessing exercise capacity. Consequently, further research is warranted to explore the long-term effects of IPC and to investigate the potential of combined exercise regimens alongside metabolite profiling.

## Data Availability

The raw data supporting the conclusions of this article will be made available by the authors, without undue reservation.
